# Discovery of stable skyrmionic state in ferroelectric nanocomposites

**DOI:** 10.1038/ncomms9542

**Published:** 2015-10-05

**Authors:** Y. Nahas, S. Prokhorenko, L. Louis, Z. Gui, I. Kornev, L. Bellaiche

**Affiliations:** 1 Physics Department and Institute for Nanoscience and Engineering, University of Arkansas, Fayetteville, Arkansas 72701, USA; 2 Department of Materials Science & Engineering and Institute of Materials Science, University of Connecticut, Storrs, Connecticut 06269, USA; 3 Laboratoire Structures, Propriétés et Modélisation des Solides, CNRS-UMR8580, Ecole Centrale Paris, 92290 Châtenay-Malabry, France

## Abstract

Non-coplanar swirling field textures, or skyrmions, are now widely recognized as objects of both fundamental interest and technological relevance. So far, skyrmions were amply investigated in magnets, where due to the presence of chiral interactions, these topological objects were found to be intrinsically stabilized. Ferroelectrics on the other hand, lacking such chiral interactions, were somewhat left aside in this quest. Here we demonstrate, via the use of a first-principles-based framework, that skyrmionic configuration of polarization can be extrinsically stabilized in ferroelectric nanocomposites. The interplay between the considered confined geometry and the dipolar interaction underlying the ferroelectric phase instability induces skyrmionic configurations. The topological structure of the obtained electrical skyrmion can be mapped onto the topology of domain-wall junctions. Furthermore, the stabilized electrical skyrmion can be as small as a few nanometers, thus revealing prospective skyrmion-based applications of ferroelectric nanocomposites.

## Abstract (web summary)

Whilst chiral magnets can support topologically protected particle-like magnetization textures called skyrmions, ferroelectric skyrmions have yet to be observed. Here, the authors use first-principles-based calculations to demonstrate the possibility of ferroelectric skyrmions in oxide nanocomposites.

## Graphical abstract

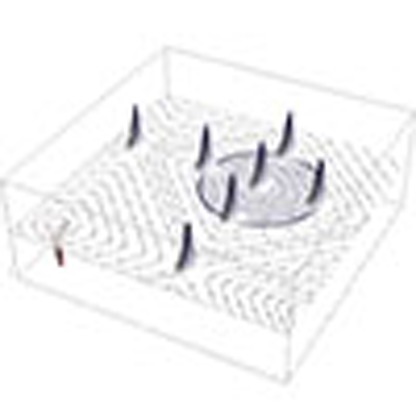

The topological invariance of nontrivial field structures such as vortices and skyrmionic configurations against fluctuations and deformations triggers considerable interest for device applications^1^,^2^. Many advances have been achieved in predicting their formation and characterizing the emergent order they subtend in magnetic systems^3^,^4^. Whereas vortex configurations, or related flux-closure configurations, have already been revealed in ferroelectrics (*FE*) within nanoscale geometries (see, for example, refs 5, 6, 7, 8, 9, 10, 11, 12, 13 and references therein), the search for a spontaneous formation of skyrmionic textures in *FE*s was somewhat hindered by the absence of intrinsic chiral interactions that are known to stabilize such configurations in non-centrosymmetric magnetic systems^14^,^15^,^16^. However, it is now unequivocal that such interactions are not a prerequisite for obtaining skyrmionic topological patterns^17^, as non-coplanar swirling field structures have been amply predicted and observed in magnets that do not meet the conditions typically deemed necessary^18^,^19^,^20^.

Recognizing in this lifted restriction a breach for probing skyrmions in ferroelectric materials, we build on the topology of the polarization field in confined geometries and show here, via the use of a first-principles-based technique, that nanoscale structures can readily become the locus of skyrmionic configuration of polarization. We also demonstrate that the field-induced skyrmionic texture is thermodynamically stable against electric and thermal perturbations. We further find that its topological charge density markedly differs from that of axisymmetric magnetic skyrmion textures in that it is broken into equally contributing fractions anchored at domain walls junctions. Our finding therefore unravels the interplay between geometry and topology in stabilizing extrinsically protected skyrmionic configuration, and brings to the fore the possibilities of extending skyrmion-based devices to ferroelectrics.

## Results

### Confined nanocomposite geometry

**Figure 1:**
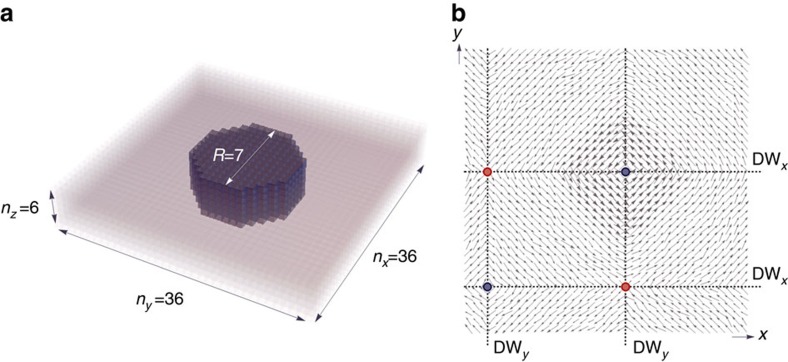
Schematic representation of the structure and dipolar configuration of the vortex state. (**a**) Schematic representation of the periodic supercell under study. The structure consists of a cylindrical BaTiO_3_ (BTO) nanowire with a radius *R* of 2.7 nm (seven lattice constant units) embedded in a SrTiO_3_ (STO) matrix with lateral sides along the [100] and [010] directions of *n*_*x*_=*n*_*y*_=36 lattice constant units, and a length *n*_*z*_=6 along the [001] pseudocubic direction. (**b**) Cross-sectional dipolar configuration of the (*V*_*xy*_|*FE*_*z*_) state characterized by a vortex pattern in the *z*-planes co-occurring with an electrical polarization along the [001] direction. Arrows correspond to the *x* and *y*-components of the electric dipoles in an arbitrary (001)-plane of the shifted periodic supercell. Blue and red circles specify the location of vortices and antivortices respectively, all occurring at the intersection of DW_*x*_ and DW_*y*_ domain walls (dashed lines) separating different configurations of the *x* and *y* components of polarization.

We choose to focus on a ferroelectric nanocomposite consisting of a cylindrical BaTiO_3_ (BTO) nanowire with a radius of 2.7 nm embedded in a SrTiO_3_ (STO) matrix in our search for electrical skyrmion. The choice of such a nanostructure is motivated by the advancement in the controlled growth of composites with tailored functionalities^21^, and the wide variety of novel behaviours they feature (note, however, that the growth of low-dimensional perovskites inside a perovskite matrix remains a difficult task to experimentally achieve). A primarily relevant topological feature of this type of nanocomposites is the occurrence of a size-induced chiral symmetry breaking, which stabilizes a vortex domain structure coexisting with a spontaneous polarization along the axial direction of the nanowire^10^. The investigated ferroelectric structure is schematized in Fig. 1a. This nanocomposite is mimicked by a 36 × 36 × 6 supercell that is periodic along the *x*, *y* and *z* axis (which lie along the pseudocubic [100], [010] [001] directions, respectively). Its properties are predicted by performing Monte Carlo simulations of the first-principles-based effective Hamiltonian scheme of ref. 22.

### Electric field treatment

The creation of an electrical skyrmion is presently achieved by a numerical procedure consisting of the following few steps. We first perform a temperature annealing under an external electric field, *E*_[001]_=10^8^ V m^−1^, applied along the pseudocubic [001] direction. On reaching 15 K, we set the field to zero and further relax the priorly obtained low-temperature configuration. The resulting relaxed configuration features a spontaneous polarization along the axial direction of the nanowire, co-occurring with a flux-closure four-domain vortex structure of the cross-sectional polarization field, in-plane pattern whereby the strength of the depolarization field is reduced^23^. More precisely, we find that the (1–3) Newnham's connectivity^24^ (BTO phase is one-dimensionally connected and the STO phase is three-dimensionally connected) of the considered nanocomposite structure hosts a polarization state possessing translational invariance along the axial direction of the wire. The polarization field thus depends only on *x* and *y* spatial coordinates, enabling a visualization of the system as consisting of two-dimensional layers (*z*-planes). The corresponding cross-sectional polarization field is shown in Fig. 1b, which displays the *x* and *y* components of the electric dipoles in an arbitrary (001)-plane of the shifted periodic supercell. This state will be referred to as (*V*_*xy*_|*FE*_*z*_) in the following, to emphasize that it has a vortex state in the *z*-planes but also possesses an electrical polarization along the [001] direction. The distribution of the latter as a function of *x* and *y* spatial coordinates is such that that local dipoles within the wire have a *z*-component around 30% larger in magnitude than that of the local dipoles located in the matrix (∼30.5 μC cm^−2^ versus ∼22.4 μC cm^−2^, respectively).

Moreover, in this (*V*_*xy*_|*FE*_*z*_) state, in addition to the central vortex whose core is confined within the nanowire, the matrix exhibits a vortex at mid-way between second-nearest neighbour wires as well as two anti-vortices at mid-way between first-nearest neighbour wires of the periodic supercell. These punctual topological defects are associated with singularities in the two-dimensional cross-sectional polarization field and are found to be anchored at the junction of DW_*x*_ and DW_*y*_ domain walls, which interpolate between domains of distinct in-plane (*x* and *y*) components of polarization (Fig. 1b). The sum of the O(2) winding numbers, each defined as a line integral measuring the change in angle of the two-dimensional cross-sectional polarization field over a closed path and giving *n*=+1 for vortices and *n*=−1 for anti-vortices^25^, yields a zero net topological charge, in accordance with Poincaré–Hopf theorem^26^.

**Figure 2:**
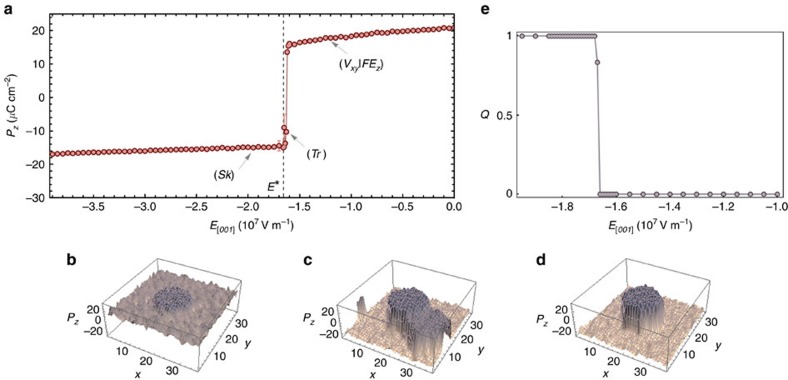
System's evolution under the application of an electric field. (**a**) Evolution of the *P*_*z*_ component of polarization with the gradually increasing external electric field 
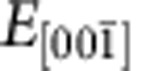
 at 15 K. Error bars indicate standard deviation, and are less than or equal to the size of the points when not visible. Arrows indicate the state (*V*_*xy*_|*FE*_*z*_) characterized by a vortex pattern in the *z*-planes co-occurring with an electrical polarization along the [001] direction as obtained for 
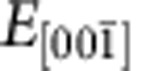
 comprised between −1.6 × 10^7^ and 0 V m^−1^, the transient state (*Tr*) obtained over the narrow range extending from −1.65 × 10^7^ to −1.6 × 10^7^ V m^−1^, and the skyrmionic state (*Sk*) obtained under E*=−1.65 × 10^7^ V m^−1^, where E* (dashed vertical line) is the threshold value of the field upon which the *z*-component of local dipoles is pointing along 
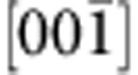
 in the matrix and along [001] in the wire. (**b**–**d**) Distribution of *P*_*z*_ within an arbitrary *z*-plane of the supercell for the (*V*_*xy*_|*FE*_*z*_) state, the transient state (*Tr*), and the skyrmionic state (*Sk*), respectively. The colour scale of **b**–**d** represents the magnitude of the out-of-plane polarization. (**e**) Dependence of the topological charge *Q* of the dipolar configurations on the external electric field.

In our search for skyrmionic configuration, we then proceed with further subjecting the (*V*_*xy*_|*FE*_*z*_) state to an 
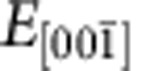
 electric field, that is, to a field pointing oppositely to (*V*_*xy*_|*FE*_*z*_)'s polarization. The evolution of the *z*-component of polarization, *P*_*z*_, with 
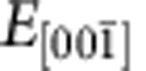
 is shown in Fig. 2a. With decreasing *E*_[001]_, one can see that *P*_*z*_ first slightly decreases before exhibiting a steep drop. Microscopical insight into the behaviour of the overall polarization is given by Fig. 2b–d, which show the distribution of *P*_*z*_ within an arbitrary *z*-plane of the supercell for three different values of 
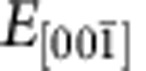
. It is therein seen that as 
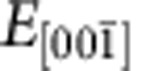
 increases, the matrix transiently develops inhomogeneity and is thus primarily accountable for the observed reduction of *P*_*z*_. After completion of this transient process, at a certain threshold value E* of the field (indicated by a vertical line in Fig. 2a), the dipolar configuration is such that the *z*-component of the electric dipoles of the matrix is anti-parallel to that of the dipoles comprised within the wire.[Fig f3]

As a result of the wire and the matrix having different switching fields, this final electric field treatment has thus prompted the nucleation of a π-wall (DW_*z*_) positioned at the interface between the wire and matrix. Let us note that for values of 
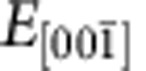
 exceeding E* and rising up to −3 × 10^8^ V m^−1^, the *z*-component of the dipoles located in the matrix slightly increases in magnitude, while the anti-aligned *z*-component of the dipoles within the wire remains quasi constant. Remarkably and as shown in Fig. 4a,b, the ensuing dipolar configuration at E* forms an electrical skyrmion (to be denoted as *Sk*) with a polarization antiparallel to E* at its center and parallel to E* at its periphery. Such skyrmionic configuration appearing at E* persists up to −3 × 10^8^ V m^−1^ and bears a radial π-modulation of the *z*-component of polarization, as well as an azimuthal modulation of the *x* and *y* components. This double modulation is characteristic of a chiral, or Bloch-like skyrmion^27^. Notably, the removal of the applied field 
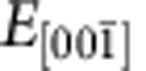
 does not alter the morphology of the (*Sk*) configuration, which indicates the stability of the obtained skyrmionic state (*Sk*). Such a finding features resemblance with magnetic systems within which occur magnetic-field-induced skyrmions that can persist after releasing the external field^17^,^28^,^29^.

### Topological characterization

**Figure 3 f3:**
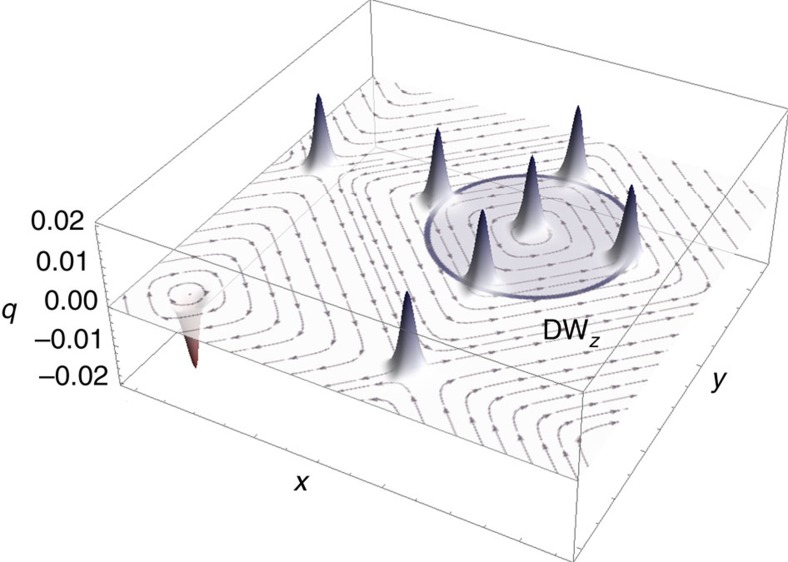
Topological charge density of the electrical skyrmion. Pontryagin density *q* in an arbitrary *z*-plane of the shifted perdiodic supercell, superimposed onto the streamlines (arrows) associated with the *x* and *y* components of the skyrmionic polarization state *(*Sk*)*. The skyrmion charge is broken into equally contributing fractions (narrow peaks) located at the junctions of domain walls. Blue (red) peaks indicate positive (negative) contributions to the total charge. The location of the nanowire is indicated by a circle, which coincides with DW_*z*_ domain wall across which the *z*-component of polarization reverses sign.

As a result of the radial π-modulation involving the *z*-component of the polarization field, an O(3) topological invariant, the Pontryagin density *q*, is required to unambiguously identify the topological nature of the obtained three-dimensional swirling field structure^17^. This invariant is defined by 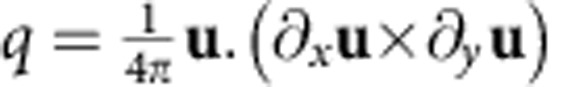, where the three-component unit vector **u** denotes the normalized local dipole moment. For a skyrmion, its surface integral over an arbitrary (001)-plane is an integer topological charge *Q* yielding the number of times **u** wraps the unit sphere^17^. Let us note that the topological density *q* can be expressed as a total derivative, and is thus intrinsically insensitive to the polarization texture details. Figure 3 provides the calculated topological density distribution with spatial derivatives being approximated using a fourth-order accurate finite difference scheme on interpolated data.

Remarkably, Fig. 3 reveals the presence of several narrow peaks located at the junctions of (DW_*x*_ and DW_*y*_), (DW_*z*_ and DW_*x*_) and (DW_*z*_ and DW_*y*_) domain walls, and equally contributing to a topological charge *Q*=1 (note that, by contrast, this invariant is trivial for (*V*_*xy*_|*FE*_*z*_), for which *Q*=0, as it is seen in Fig. 2e). This finding, which we further cross-checked through the method of topological charge computation of ref. 30, attests to the topological nature of the obtained skyrmionic configuration. It also demonstrates that its broken topological structure can be mapped onto the topology of domain-wall junctions, as a genuine consequence of the considered nanocomposite geometry. A distinctive feature of the electrical (*Sk*) is thus its four-fold symmetric topological charge density, markedly differing from that of axisymmetric magnetic skyrmion textures and other topological objects. Moreover, the geometry of the system enables obtaining an electrical skyrmion whose size can be as small as a few nanometers whereas magnetic skyrmions non-arising from Dzyaloshinskii–Moriya interaction extend on significantly larger scales^17^,^18^,^29^, thereby widening the scope and capabilities of future skyrmion-based applications.

### Assessment of the skyrmionic state's stability

**Figure 4:**
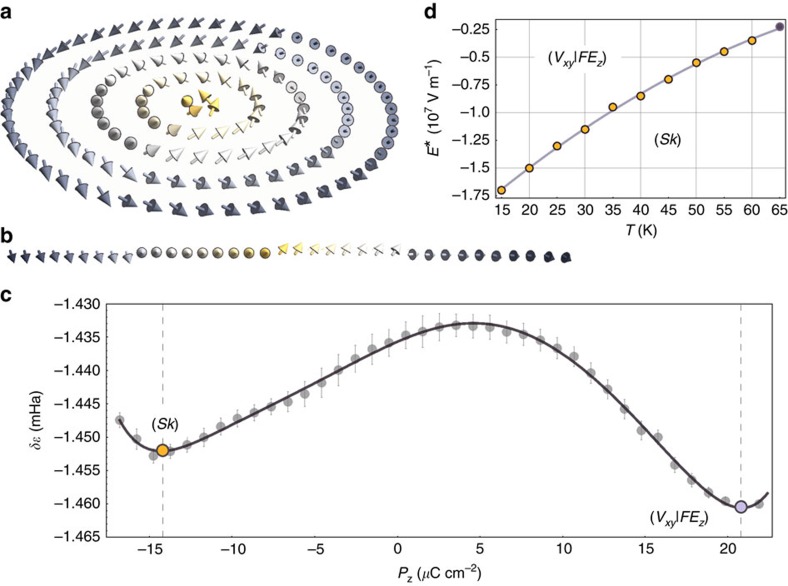
Stability of the skyrmionic state. (**a**) Polarization field configuration within the relaxed skyrmionic state (*Sk*) as obtained from the simulation at 15 K. (**b**) Local dipoles plotted along a line passing through the skyrmion's core and parallel to the *x* coordinate axis. It is seen that the tips of the dipoles describe a circle in the plane perpendicular to the line, thus indicating the variation of the *y* and *z* components along this line. The vectors are coloured according to their *z*-component in **a**,**b**. (**c**) Dependence of the internal energy *δɛ* on the *P*_*z*_ component of polarization showing the crossover between two stable minima: the one corresponding to the (*V*_*xy*_|FE_*z*_) state characterized by a vortex pattern in the *z*-planes co-occurring with an electrical polarization along the [001] direction, and the one associated with the skyrmionic texture (*Sk*). Error bars indicate s.d. (**d**) Temperature dependence of the estimated threshold external electric field E* needed to drive the system towards the skyrmion state.

Furthermore and as above demonstrated, the (*Sk*) state is at the least metastable. To bridge the topologically distinct (*V*_*xy*_|*FE*_*z*_) and (*Sk*) states and estimate their relative stability, we have performed a Metropolis Monte Carlo sampling of the transition paths connecting them in configurational space. Different Markov chains allowed to gather statistics on the pathways accessible under the application of E* on the (*V*_*xy*_|*FE*_*z*_) configuration. Averaging the energies associated with different trajectories parametrized by the *P*_*z*_ component of polarization yielded the internal energy profile shown in Fig. 4c. It appears clearly that the obtained (*Sk*) is metastable with a local minimum only 7 μHa higher than that of the (*V*_*xy*_|*FE*_*z*_) state. While both the (*V*_*xy*_|*FE*_*z*_) and (*Sk*) states are stabilized by long-range dipolar interactions, we find that the difference in their energy mainly originates from the π-wall energy formation cost encoded in the short-range interaction. The topological stability of the (*Sk*) state is reflected in the finite energy barrier which protects it against spontaneous decay. This energy barrier (∼0.02 mHa) underpins the first-order nature of the transition associated with the abrupt partial switching process (Fig. 2a), in that the skyrmionic structure cannot be continuously deformed into that of the (*V*_*xy*_|*FE*_*z*_) state.

We also probe the thermal stability of the (*Sk*) state by increasing the temperature. At moderate temperatures, thermal fluctuations do not destabilize the (*Sk*) state, which remains robust up to 65 K. Specifically, thermally activated excitations in the form of confined and self-compensating O(2) punctual defects (vortex-antivortex bound pairs) induce homotopical configurations preserving the topological texture of the (*Sk*) state. This finding is consistent with the range of stability shown in Fig. 4d, which provides the temperature dependence of the estimated threshold external electric field E* needed to drive the system towards the skyrmion state. It is seen that E* decreases in magnitude as the temperature is increased. The transition line between the (*Sk*) and the (*V*_*xy*_|*FE*_*z*_) states terminates at a finite temperature and field (65 K and −0.24 × 10^7^ V m^−1^), on which the topological protection is no longer in effect. At this termination point, thermal and electrical excitations endow the system with a sufficiently high energy to overcome the topological barrier. The (*Sk*) thermal stability range can be enhanced by substituting the BTO material composing the wire with a higher-T_C_ ferroelectric, while conserving a discrepancy between the wire and matrix polarizabilities.

We further appraised the skyrmion stability by subjecting it to a gradually increasing *E*_[111]_ electrical field oriented along the [111] direction at 15 K. While under a certain threshold of *E*_[111]_ the excited configuration relaxes back to the initial (*Sk*) state, for strong enough magnitude, the induced nonequilibrium state converts into an homogeneous polar rhombohedral state (to be denoted by (*Rh*)) of higher energy (−1.45 mHa). This metastable (*Rh*) state consists of electric dipoles pointing along the [111] direction in both the wire and the matrix. We find that the energy barrier associated with this annihilation pathway (∼0.05 mHa) is higher than the one connecting the (*Sk*) and the (*V*_*xy*_|*FE*_*z*_) states. The reason behind the difference in height of the barriers can be approached through topological considerations; while a transition from the (*Sk*) state to the topologically trivial (*Rh*) state requires disentangling the former by unwinding its two modulations, the transition from the (*Sk*) state to the (*V*_*xy*_|*FE*_*z*_) state involves releasing the π-modulation, thereby only affecting the *z*-component of polarization while conserving the cross-sectional configuration of polarization and its domain-wall-pinned O(2) punctual defects.

## Discussion

Inquiring into the dependence of the topological (*Sk*) texture on the geometry of the structure, we performed additional simulations where the effect of the radius of the nanowire, its shape and the length *n*_*z*_ along the [001] pseudocubic direction of the supercell were assessed. We found that the results are not affected by neither *n*_*z*_ (over the range *n*_*z*_=6 to *n*_*z*_=10) nor the shape (square versus circle cross-sections) of the nanowire. Varying the size of the wire yielded a non-monotonic dependence of E* on the radius *R* of the cylindrical wire. With increasing radii of BaTiO_3_ nanowires, E* was found to increase, merely indicating the relatively empowered influence of the wire on the matrix as a result of its enlargement. Moreover, we found that considering a rectangular array of cylindrical wires within the supercell endowed the resulting skyrmion lattice with helicity 
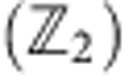
 degrees of freedom (the circulation direction of the cross-sectional polarization around the skyrmion core); depending on the inter-wire distances, the interaction between skyrmions led to either a single-chirality (phase-locking) pattern or to an alternate arrangement of skyrmionic chiralities within this lattice. We also note that skyrmion-like configurations should exist in other geometries than the nanocomposite structure herein investigated, provided that similar physics comes into play^31^. Moreover, free charges and structural defects can screen or modify depolarizing field and thus affect the formation of electrical skyrmions. However, we are confident that these latter topological defects will soon be observed based on the fact that other depolarizing-field-related dipolar patterns have already been experimentally seen in some ferroelectric systems (see, for example, refs 9, 10, 11, 12, 13).

In conclusion, we have shown that ferroelectric nanocomposites can accommodate a skyrmionic configuration as a stable state of polarization. Our study brings to the fore the interplay between geometry and topology in providing extrinsic topological protection. Finally, analysing the (*Sk*) topological properties, we found that its topological charge density is broken into fractions pinned at domain walls junctions, thereby singling out electrical skyrmions from topological objects with axisymmetric topological charge densities.

## Methods

### Effective Hamiltonian

The effective Hamiltonian employed in this study is that of ref. 22. Its total internal energy has the form:


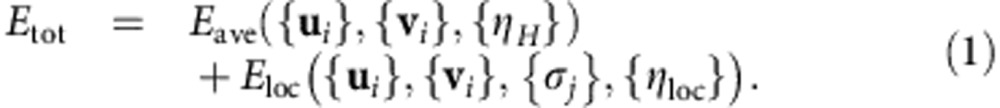


The first energy term, *E*_ave_, depends on the local soft mode **u**_*i*_ centred on the Ti-sites of the 5-atom unit cell *i* and directly proportional to the electric dipole moment of the corresponding unit cell, the dimensionless displacement variables {**v**_*i*_} defined at the cell corners and entering in the calculation of the inhomogeneous strain tensor components of the cell *i*^32^,^33^, and the homogeneous strain tensor {*η*_*H*_}, which allows the simulations to account for the change in size and shape of the supercell^32^,^33^. *E*_ave_ consists of five parts: a local-mode self-energy, a long-range dipole-dipole interaction, a short-range interaction between soft modes, an elastic energy, and an interaction between the local modes and local strain^32^,^33^. Its parameters are fitted from first-principles calculations performed on a uniform virtual 〈*A*〉TiO_3_ system that averages the potentials of the pure parent compounds^34^,^35^ to model (Ba_0.5_Sr_0.5_)TiO_3_ solid solutions. The second energy term, *E*_loc_, involves the {*σ*_*j*_} parameters and {*η*_loc_}, which respectively correspond to the set of variables {*σ*_*j*_} characterizing the atomic distribution of the mixed A-sublattice^36^, with *σ*_*j*_=+1 or −1 corresponding to the presence of either Ba or Sr atom at the A-lattice site *j*, and the local strain {*η*_loc_} stemming from the difference in ionic radii between Ba and Sr atoms (which is 
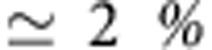
). This second energy term *E*_loc_ stands as a perturbative correction to the virtual crystal approximation, as it accounts for the real nature of the A-site atom and its effect on the local soft modes and the inhomogenuous strain tensor, as well as it includes corrections pertaining to the strain induced by the size difference between Ba and Sr ions^22^. The parameters entering in *E*_loc_ are derived from first principles. The nanocomposite herein considered is stress-free, which allows the matrix, interface and wires to adopt different lattice constants, thereby modelling a (realistic) inhomogeneous strain. The Monte Carlo simulations were performed using up to 50,000 sweeps.

It is worth mentioning that the above described first-principles-based effective Hamiltonian scheme^22^ has been shown to accurately reproduce various (static and dynamical) properties of different disordered or chemically ordered (Ba,Sr)TiO_3_ systems^22^,^37^,^38^,^39^,^40^. One example includes Curie temperatures and phase diagrams^22^ of (Ba,Sr)TiO_3_ materials (BST)^39^. For instance, our effective Hamiltonian gives transition temperatures of 385 K (from cubic to tetragonal), 280 K (from tetragonal to orthorhombic) and 230 K (from orthorhombic to rhombohedral) in BaTiO_3_ bulk, which compares rather well with the experimental values of 400, 280 and 180 K^22^. Other examples demonstrating the accuracy of this effective Hamiltonian is the subtle temperature-gradient-induced polarization^40^, and the existence of two modes (rather than a single one as previously believed for a long time) contributing to the GHz–THz dielectric response of BST compounds^39^.

Notably, nanocomposites such as the one considered in the present study and consisting of BTO nanowires embedded in a STO matrix, were predicted to exhibit a spatially modulated field structure from which a variety of striking phenomena^41^,^42^,^43^ originates. Let us note that the simulated matrix does not rigorously correspond to pure STO but rather coincides with Ba_1−*x*_Sr_*x*_TiO_3_ having 85% of strontium because of some limitations related to the employed effective Hamiltonian (in particular, this effective Hamiltonian predicts a paraelectric-to-ferroelectic transition of pure STO at a Curie temperature that experimentally corresponds to the one of Ba_0.15_Sr_0.85_TiO_3_).

A final remark pertains to the magnitude of electric fields reported in this study. Generally, effective Hamiltonian schemes have a tendency to overestimate electric experimental fields by a factor of about 25 with respect to experiments^44^. However, a field of 10^9^ V m^−1^ has been applied to some nano-structures in recent measurements^45^.

## Additional information

**How to cite this article:** Nahas, Y. *et al*. Discovery of stable skyrmionic state in ferroelectric nanocomposites. *Nat. Commun.* 6:8542 doi: 10.1038/ncomms9542 (2015).
